# International IVIg prescription patterns in idiopathic inflammatory myopathies: real-world insights from the MyoNet survey

**DOI:** 10.1016/j.ero.2026.02.012

**Published:** 2026-03-03

**Authors:** Kastriot Kastrati, Latika Gupta, James B Lilleker, Choon-Guan Chua, Thomas Khoo, Ho So, Paul New, Helga Lechner-Radner, Hector Chinoy, MyoNet

**Affiliations:** 1 Division of Rheumatology, Department of Internal Medicine III, Medical University of Vienna, Vienna, Austria; 2 Department of Rheumatology, Royal Wolverhampton Hospitals NHS Trust, Wolverhampton, UK; 3 School of Infection, Inflammation and Immunology, College of Medicine and Health, University of Birmingham, Birmingham, UK; 4 Muscle Disease Unit, Manchester Centre for Clinical Neuroscience, Northern Care Alliance NHS Foundation Trust, Manchester Academic Health Science Centre, Manchester, UK; 5 Department of Rheumatology, Allergy and Immunology, Tan Tock Seng Hospital, Singapore, Singapore; 6 Rheumatology Unit, Royal Adelaide Hospital, Adelaide, SA, Australia; 7 Rheumatology Unit, Southern Adelaide Local Health Network, Adelaide, SA, Australia; 8 Faculty of Health and Medical Sciences, University of Adelaide, Adelaide, SA, Australia; 9 Department of Medicine and Therapeutics, The Chinese University of Hong Kong, Hong Kong SAR; 10 Centre for Musculoskeletal Research, Division of Musculoskeletal & Dermatological Sciences, Faculty of Biology Medicine and Health, The University of Manchester, Manchester, UK; 11 Department of Rheumatology, Salford Royal Hospital, Northern Care Alliance NHS Foundation Trust, Manchester Academic Health Science Centre, Salford, UK; 12 Division of Musculoskeletal and Dermatological Sciences, Faculty of Biology, Medicine and Health, The University of Manchester, Manchester, UK

## Abstract

**Objectives:**

Intravenous immunoglobulin (IVIg) is increasingly used in idiopathic inflammatory myopathies (IIMs), especially in cases with complex clinical presentations or resistance to conventional immunosuppressants. However, real-world prescription patterns remain poorly defined and are likely influenced by regional, institutional, and economic factors.

**Methods:**

A 31-item, English-language e-survey was distributed through the MyoNet registry members after pilot testing and expert review. The survey assessed institutional IVIg treatment practices in IIM, comprising indications, dosing strategies, perceived limitations, and adverse events (AEs). Regional comparisons were performed, and socioeconomic metrics including Human Development Index (HDI) and national gross domestic product (GDP) per capita were correlated with reported barriers.

**Results:**

Across 68 centres in 27 countries, IVIg was most frequently used in dermatomyositis (85.3%), immune-mediated necrotising myopathy (82.4%), and antisynthetase syndrome (66.2%). Dysphagia, refractory disease, and rapidly progressive courses were the leading indications. IVIg was predominantly used as second- or third-line treatment and commonly administered alongside glucocorticoids and steroid-sparing immunosuppressants. AEs were frequent (76.5% of centres), most commonly headaches and infusion reactions. Non-European centres more often used IVIg in seropositive patients, interstitial lung disease, and when other immunosuppressants were contraindicated. Access to IVIg significantly correlated with national development and wealth: cost and availability were more restrictive in countries with lower HDI (cost: r = –0.26, *P* = .03; availability: r = –0.31, *P* < .01) and lower GDP (cost: r = –0.27, *P* = .03; availability: r = –0.34, *P* < .01).

**Conclusions:**

This international survey provides a current overview of real-world IVIg use in IIM. Our findings support the need for harmonised treatment guidance to promote equitable access across healthcare systems.

**WHAT IS ALREADY KNOWN ON THIS TOPIC**

• Intravenous immunoglobulin (IVIg) is an established therapy in dermatomyositis following the ProDERM (Progress in DERMatomyositis) trial, but its use in other idiopathic inflammatory myopathy (IIM) subtypes and organ manifestations is supported by limited evidence.

**WHAT THIS STUDY ADDS**

• This study provides the first multinational, real-world overview of institutional IVIg use across the full IIM spectrum, beyond controlled trial settings.
• IVIg is frequently prescribed for indications with limited or absent evidence, including dysphagia, interstitial lung disease, cardiac and gastrointestinal involvement, and refractory disease across multiple IIM subtypes.
• Indication patterns vary regionally, reflecting differences in disease phenotype prioritisation, prior immunosuppressive exposure, and healthcare system constraints rather than evidence alone.
• Socioeconomic disparities influence access to IVIg, with lower-income countries facing greater cost and availability barriers.

**HOW THIS STUDY MIGHT AFFECT RESEARCH, PRACTICE OR POLICY**

• By identifying where IVIg is most used despite limited evidence, this study may highlight priority areas for future clinical trials and observational research in IIM.
• The findings support the need for harmonised international guidance that acknowledges real-world practice while addressing evidence gaps and socioeconomic barriers.

Alt-text: Unlabelled box dummy alt text

## INTRODUCTION

Idiopathic inflammatory myopathies (IIMs) are recognised as a heterogeneous group of autoimmune diseases primarily affecting skeletal muscle, encompassing dermatomyositis (DM), juvenile DM (JDM), antisynthetase syndrome (ASyS), immune-mediated necrotising myopathy (IMNM), overlap myositis (OM), inclusion body myositis (IBM), cancer-associated myositis (CAM), and polymyositis (PM) [1,2]. Although these entities share core features, they differ considerably in clinical phenotype, organ involvement, and treatment response [3]. Traditionally, high-dose corticosteroids have been the first-line treatment for IIM, usually combined with steroid-sparing immunosuppressants such as methotrexate, azathioprine, or mycophenolate mofetil [4]. However, these regimens are not universally effective, and a substantial subset of patients either fail to achieve adequate disease control or develop adverse events (AEs) that limit their continuation.

Intravenous immunoglobulin (IVIg) has emerged as a key therapeutic modality in such cases, particularly in clinically complex scenarios where conventional immunosuppressive strategies are either ineffective, contraindicated, or poorly tolerated [[5], [6], [7], [8], [9]]. IVIg is often employed off-label and without formal guidance. However, the body of evidence was significantly strengthened by the publication of the ProDERM (Progress in DERMatomyositis) trial, a landmark phase III randomised study demonstrating the efficacy and safety of IVIg in patients with active DM [10], which led to regulatory approval for DM in both Europe and the United States [11]. The trial’s exclusive focus on DM leaves important questions unanswered regarding the use of IVIg in IIM subtypes beyond DM, such as IMNM or ASyS. Further, it is unclear how IVIg is used in real-world clinical practice outside of controlled trials and how factors such as local reimbursement policies, drug availability, and infrastructural capacities impact its use. Key aspects such as the timing of initiation, duration of therapy, requirements for weaning, and dosing regimens are influenced by multiple factors such as disease severity, subtype, and individual treatment response [12,13]. This variability suggests a discrepancy between trial-based evidence and real-world practice. A recent patient-reported survey by Ziade et al and the COVID-19 vaccination in autoimmune diseases (COVAD) group among 1418 individuals with IIM confirmed significant treatment inequities and showed that access to advanced therapies is largely limited to high-income nations [14].

Despite the high cost and limited global availability of IVIg, there is no consolidated understanding of how physicians use IVIg in routine care across clinical settings, countries, and regions. Recognising this unmet need, MyoNet initiated a comprehensive, international physician survey to systematically document and analyse current IVIg prescription practices in IIM. The primary objective was to understand and confirm physicians’ perspectives on current treatment practices such as common indications, therapeutic approaches, and rationale behind treatment decisions as well as practical barriers and obstacles related to IVIg use. By doing so, this initiative aims to support consistent guidance and promote equitable access to IVIg therapy across different healthcare systems.

## METHODS

### Survey design

A 31-item survey was developed by the study authors to evaluate clinical decision-making, treatment patterns, and practical experiences. The questionnaire included domains such as indications for IVIg initiation, dosing strategies, treatment duration, limitations during usage, response assessment, and observed AEs. Additional sections addressed clinical scenarios and disease phenotypes in which IVIg is preferentially applied, as well as institutional experience across IIM subtypes. Most items were multiple-choice questions with single- or multiple-answer options, supplemented by rating scales and percentage sliders to assess frequency or relevance. Multiple-choice questions with multiple responses permitted were incorporated where applicable, and several items included an ‘Other (please specify)’ option to allow for free input beyond predefined categories. At the end of the survey, participants were invited to provide information on demographics, including medical specialty, country/region of practice, institutional setting, years of clinical practice within their area of specialisation (including years in training), and additional comments via a free-text window.

The survey was developed in a multistep process covering expert input of the MyoNet consortium, which is an international, multidisciplinary network of clinicians and researchers dedicated to IIM, to ensure face validity and clinical relevance. The questionnaire was subsequently implemented in the SurveyMonkey platform and distributed via an email list to all registered MyoNet members. Participation was limited to 1 designated responder per centre, and responders were encouraged to complete the survey based on institutional practices rather than individual cases. The selection of the responder was performed locally by each centre. Responses were collected between January 23, 2025 and March 23, 2025.

As this was a physician-based survey without the collection of individual patient-level data, no formal ethical approval was required under national or institutional regulations. Participation was anonymous, and all responses were analysed in aggregate. A complete version of the survey is provided as a supplementary file ‘questionnaire’.

### Statistical analysis

Survey responses were excluded from the analysis if fewer than 60% of questionnaire items were completed. Submissions meeting this completion threshold were included in the analysis. All statistical analyses were performed using R software (version 4.5.1; R Foundation for Statistical Computing). The package ‘ggplot2’ was used to generate all graphical outputs. For figures involving multiple colour gradients, the ‘viridis’ colour palette was selected. Categorical variables were presented as absolute numbers and percentages. Continuous variables were summarised using mean and SD or median and IQR, depending on data distribution. Normality was assessed using the Shapiro-Wilk test and visual inspection of distribution plots. Group comparisons, particularly between European and non-European centres, were conducted to evaluate differences in IVIg prescription practices. For categorical variables, the chi-square independence test or Fisher’s exact test was applied as appropriate. For continuous variables, comparisons were performed using an independent samples t-test for normally distributed data, or the Mann-Whitney U test in the absence of normality.

To explore the relationship between economic context and country/region-specific responses regarding IVIg availability and treatment cost as limiting factors, we correlated the grading of limitations with the respective country’s/region’s Human Development Index (HDI), a composite indicator including life expectancy, education, and standard of living, as well as gross domestic product (GDP) per capita (in US-Dollar, $USD). For each participating centre, self-reported scores for availability and cost were matched to HDI and GDP metrics retrieved from publicly accessible sources (if available), which were the United Nations Development Programme (https://hdr.undp.org/data-center/human-development-index#/indicies/HDI) for HDI and the World Bank database (https://data.worldbank.org/) for GDP, reflecting the most recent estimates available for the year 2023 (accessed on May 24, 2025). Correlations were calculated using Spearman’s rank correlation coefficient. Statistical significance was defined as a *P* value <.05.

## RESULTS

### Centre characteristics

**Figure 1:**
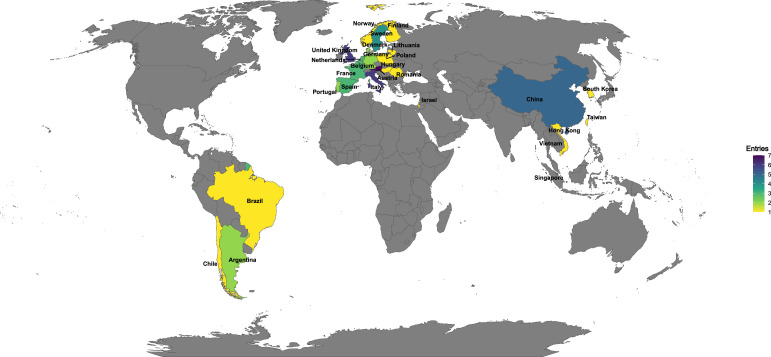
Geographic distribution of participating centres. Countries/regions are portrayed according to the number of responding centres, with yellow tones indicating fewer entries and violet tones representing a higher number of centres (1-7). Disclaimer: This map is provided for illustrative purposes only and does not reflect the views of the authors regarding geopolitical borders.

A total of 80 responses were received, of which 12 were excluded because of a complete lack of data entry. The final analysis included 68 completed surveys from centres actively managing patients with IIM. Most respondents were specialised in rheumatology (72.1%), followed by internal medicine (14.7%), neurology (5.9%), paediatrics (4.4%), with dermatology, and pulmonology each represented by 1 centre (1.5%). In terms of clinical experience, 41.2% reported 11 to 20 years of practice, and 26.5% had more than 20 years of professional experience in their respective fields. Among the included centres, 27 countries/regions were represented in the final dataset. The highest number of participating centres came from Austria (n = 7), followed by Italy (n = 6) and the United Kingdom (n = 6). A visual overview of geographic distribution is shown in Figure 1. Regarding patient volumes, 26.5% of centres reported actively caring for 26 to 50 patients with IIM, and 23.5% managed between 51 to 100 patients. In terms of IVIg therapy, the majority of centres (72.1%) reported currently treating fewer than 10 patients, whereas only 2.9% indicated treating more than 25 patients with IVIg. A detailed overview of participating countries/regions and centre-specific characteristics is provided in Supplementary Table S1.

### Clinicoserologic profile and treatment history of patients at the time of IVIg initiation

Across the 68 participating centres, IVIg use was most frequently reported for DM, with 85.3% of centres indicating IVIg use in this subtype, followed by IMNM (82.4%). Other subtypes for which IVIg use was commonly reported included ASyS (66.2%), CAM (55.9%), PM (51.5%), and OM (48.5%). IVIg use was less frequently reported for JDM (19.1%) and IBM (11.8%). A graphical overview of centre-reported IVIg use across IIM spectrum is presented in Supplementary Figure S1.

Regarding patient profiles at the time of IVIg initiation, respondents provided centre-level estimates of patient characteristics. On average, 73.4% of IVIg-treated patients were reported to be seropositive for myositis-specific antibodies or myositis-associated antibodies (MSAs/MAAs), whereas approximately 18.5% were seronegative. Severe muscle involvement was the most frequently reported clinical feature (58.8%), followed by treatment-refractory disease (49.8%), rapidly progressive disease course (35.4%), contraindications to conventional immunotherapies (27.1%), and prominent skin or pulmonary manifestations (24.9% and 23.6%, respectively) (details are shown in Supplementary Table S2).

Overall, use of IVIg as first-line therapy was reported with a mean frequency of 22.1% ± 22.4% (median: 15%, IQR: 5.0%-30%), which indicated that IVIg was more commonly employed as a second- or third-line option in clinical practice. Before IVIg initiation, most patients had been previously treated with at least 1 or 2 disease-modifying antirheumatic drugs: 23.5% of the centres reported starting IVIg after failure of 1 agent, and 29.4% after 2. Commencing IVIg after more than 3 remedies had failed was uncommon. The most frequently used agents preceding IVIg included methotrexate (80.9%), mycophenolate mofetil (69.1%), and rituximab (47.1%), followed by azathioprine (44.1%), and calcineurin or mammalian target of rapamycin (mTOR) inhibitors (29.4%). Concomitant immunosuppressive treatment alongside IVIg was common, with an average of 80.7% ± 24.5% of patients receiving additional agents. As such, glucocorticoids were coadministered by 88.2% of the centres. Additional agents frequently used in parallel, as reported at the centre level, included methotrexate (79.4%), mycophenolate mofetil (70.6%), rituximab (48.5%), or azathioprine (39.7%). An overview of pretreatment and concomitant immunosuppressive regimens as reported by centres is provided in Supplementary Table S3.

### Disease gestalt and treatment lines for IVIg use

Most centres (88.2%) indicated that IVIg was typically initiated in cases refractory to other immunosuppressants. Rapidly progressive disease (76.5%), dysphagia (73.5%), and contraindications to conventional immunosuppressants (67.6%) were also commonly selected indications. IMNM (63.2%) and CAM (52.9%) were frequently named as specific disease contexts for IVIg use, whereas reproductive considerations (pregnancy, conception, breastfeeding: 27.9%) and steroid-sparing aspects (26.5%) played a role as well. In addition, most centres reported initiating IVIg primarily in patients with moderate (44.1%) to severe (51.5%) disease activity, which emphasises its role as an escalation strategy in more active disease stages (Supplementary Table S4 for full details).

**Figure 2:**
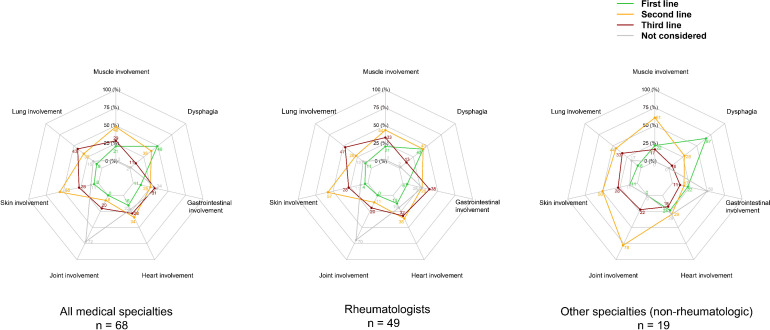
Treatment line stratification of IVIg use for specific clinical manifestations in IIM across all medical specialties (left), rheumatologists (middle), and nonrheumatologists (right). Reported treatment line for IVIg use across different clinical manifestations in IIM is stratified by all specialties (n = 68), rheumatologists (n = 49), and other specialties (n = 19). The values indicate the proportion of respondents/centres categorising IVIg as first- (green line), second (orange line)-, or third line (red line) therapy, or stating that IVIg is not considered an appropriate treatment option for the specific manifestation (grey line). IIM, idiopathic inflammatory myopathy; IVIg, intravenous immunoglobulin.

To gain further insights into how IVIg is integrated across different treatment stages for concrete muscular or extramuscular manifestations, centres were asked to stratify IVIg use as first-, second-, or third-line therapy across various clinical domains. Aggregated data for all responding centres revealed that dysphagia was the predominant indication for first-line IVIg use, followed by muscle involvement, which was broadly acknowledged across medical specialties as a key indication. Notably, for skin involvement, lung manifestations, and gastrointestinal complications (eg, gastroparesis, intestinal dysmotility/pseudo-obstruction, vasculitic or ischaemic complications), IVIg was more commonly employed as a second-line treatment. A stratified comparison between rheumatologists (n = 49) and nonrheumatologists (n = 19) revealed overlapping trends, but also distinct preferences. Rheumatologists more frequently considered IVIg as a second-line option for manifestations such as dysphagia (43% vs 28%) and cardiac involvement (36% vs 29%). In contrast, clinicians from other specialties more often prioritised IVIg as a second-line treatment for joint involvement (78% vs 11%) and pulmonary involvement (44% vs 28%). A detailed depiction of treatment lines across specialties is provided in Figure 2.

### IVIg administration practices and treatment response assessment

Most centres (63.2%) reported calculating IVIg doses based on actual body weight, whereas 27.9% referred to ideal body weight. The most common total dose administered was 2 g/kg body weight (used by 75% of centres), followed by 1 g/kg (14.7%). Administration intervals were highly standardised, with 82.4% of centres applying a 4-week cycle. The mean reported duration of an IVIg treatment course was 5.9 ± 5.2 months (median: 4.5 months, IQR: 3.0-6.0). Evaluation of treatment efficacy typically occurred after 6 weeks (41.2% of centres) or 12 weeks (51.5%) of treatment, with only a minority of centres assessing outcomes later. After reaching disease control, the most common strategy was extending the treatment interval (42.6%), often accompanied by dose reduction (25.0%). A subset of centres reported applying subcutaneous immunoglobulin formulations (14.7%). To assess clinical response to IVIg, centres rated the use of various tools on a 0 to 5 rating scale (0 = not assessed, 5 = integral component of monitoring). The most frequently applied parameters included muscle enzyme levels (mean score 4.5 ± 0.8) and steroid dose reduction (4.1 ± 1.2), followed by patient-reported outcomes (3.4 ± 1.5). In contrast, formal instruments such as the American College of Rheumatology (ACR) and the European Alliance of Associations for Rheumatology (EULAR) Total Improvement Score or timed function assessments played a lesser role (both with mean ratings <2) (Supplementary Table S5 for detailed results). Key characteristics of IVIg use across participating centres are summarised in Table 1.

**Table 1:** Key characteristics of IVIg use across participating centres

Domain	Result
Main indication	Refractory disease (88.2%)
First-line use	Median 15% (IQR 5-30)
Standard dose	2 g/kg body weight (75.0%)
Cycle interval	4 wk (82.4%)
Main response parameter	Muscle enzymes (mean score 4.5)

IVIg, intravenous immunoglobulin.

Summary of the most commonly reported indications, treatment line, dosing practices, and response assessment parameters for IVIg therapy, based on centre-level survey responses. Values represent the proportion of centres reporting the respective practice, unless otherwise specified.

### AEs associated with IVIg therapy

The occurrence of any AEs potentially attributable to IVIg was reported by 76.5% (n = 52) of participating centres. The most cited events at the centre level included headaches (61.8%), infusion reactions such as allergic or anaphylactic responses (42.6%), fever (33.8%), and nausea (26.5%). Serious complications such as thromboembolic events (23.5%), aseptic meningitis (22.1%), and cerebrovascular events (4.4%) were also reported. Other notable symptoms included chills (19.1%), myalgia (14.7%), and vomiting (8.8%). Cerebrovascular events such as stroke or cerebral infarction were infrequently reported (4.4% of the centres). A detailed overview of reported AEs is presented in Table 2.

**Table 2:** Reported adverse events associated with IVIg use

Adverse events specified	Percentage (%)
Total no. (centres/patients)	68 centres
Headaches	61.8
Infusion reaction (allergic, anaphylactic)	42.6
Fever	33.8
Nausea	26.5
Thromboembolism (deep-vein thrombosis, pulmonary embolism)	23.5
Aseptic meningitis	22.1
Chills	19.1
Myalgia	14.7
Vomiting	8.8
Cerebrovascular accident/cerebral infarction	4.4
Other^a^	7.4

IIM, idiopathic inflammatory myopathy; IVIg, intravenous immunoglobulin; no./n, number of centres or patients; %, percentage of centres/patients.

Values indicate the proportion of centres (n = 68) that have ever observed the respective adverse event in patients with IIM treated with IVIg.

a Other: n = 2 myocardial infarction, n = 1 serum sickness, n = 1 other immune disease, n = 1 neutropenia.

### Global differences in IVIg practice: Europe compared with other regions

Regional comparisons showed notable differences in IVIg use and treatment approaches between European (n = 47) and non-European centres (n = 21). Centres outside Europe reported more frequent use of IVIg in patients with ASyS (85.7% vs 57.4%, *P* = .04). Consistently, IVIg was more commonly applied in non-European countries/regions for patients with positive MSA/MAA (80.8% vs 70.0%) and interstitial lung disease (ILD; 31.5% vs 20.1%). In addition, contraindications to other immunosuppressive therapies were more frequently present among IVIg-treated patients in non-European centres (42.2% vs 19.4%, *P* = .01), as were concomitant infections (76.2% vs 44.7%, *P* = .03). In terms of prior treatment, non-European centres reported significantly more frequent use of calcineurin inhibitors or mTOR inhibitors (66.7% vs 12.8%, *P* < .01) and cyclophosphamide (47.6% vs 21.3%, *P* = .04) before IVIg. European centres more frequently reported concomitant use of methotrexate alongside IVIg (87.2% vs 61.9%, *P* = .02). Regarding adjustments after disease control, European centres more often reported dose reduction by 50% (29.8% vs 4.8%, *P* = .03), whereas non-European centres tended to maintain the initial regimen (42.9% vs 6.4%, *P* < .01).

Differences also emerged regarding limitations to IVIg administration, with cost being a greater limiting factor in non-European countries/regions (4.0 ± 1.4 vs 2.7 ± 1.9 on a scale from 0 [not a limitation] to 5 [major limitation]; *P* < .01). Conversely, availability appeared to be a more relevant barrier in European centres (2.2 ± 2.0 vs 1.0 ± 1.5; *P* = .02). European centres were also more likely to follow national/regional guidelines or commissioning policies (40.9% vs 4.8%, *P* < .01). For a full overview of region-specific data and its comparisons, see Supplementary Table S6.

### Health-economic barriers to IVIg therapy: impact of national wealth

**Figure 3:**
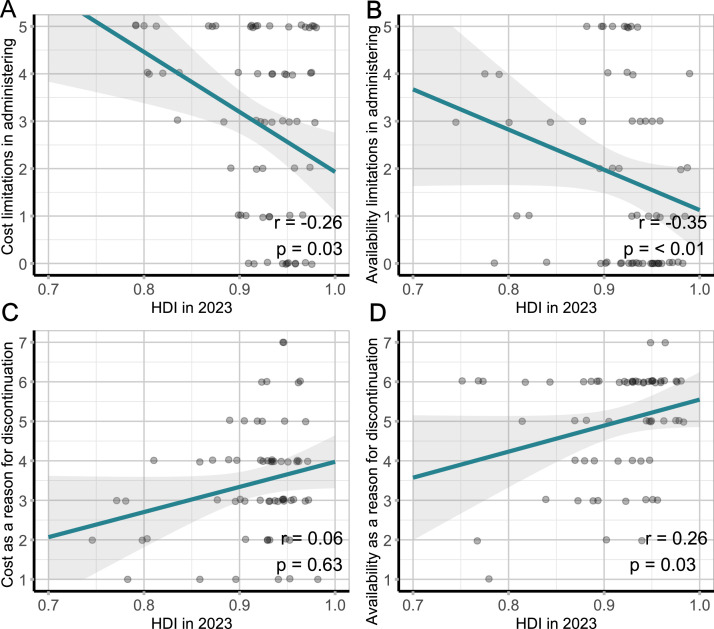
Correlation between HDI and perceived barriers to IVIg use. Spearman’s rank correlation coefficients (r) and *P* values (*P*) are shown; lower scores indicate greater limitation or higher importance. (A) Cost as a limitation to initiating IVIg (rating 0-5; r = –0.26, *P* = .03). (B) Availability as a limitation to initiating IVIg (rating 0-5; r = –0.35, *P* < .01). (C) Cost as a reason for discontinuation (rating 1-7; r = 0.06, *P* = .63). (D) Availability as a reason for discontinuation (rating 1-7; r = 0.26, *P* = .03). Rating scales: 0-5 = limitation severity (0 = none, 5 = major) for (A/B); 1-7 = importance of discontinuation reason (1 = most, 7 = least) for (C/D). HDI, Human Development Index; IVIg, intravenous immunoglobulin; Spearman’s r, Spearman rank correlation coefficient.

We analysed correlations between HDI as well as GDP and reported limitations and discontinuation reasons across centres. Figure 3A shows a significant inverse correlation between HDI and cost as a limitation to initiate IVIg (Spearman’s r = –0.26, *P* = .03). Thus, centres in lower-HDI countries rated cost as a more severe obstacle (higher values on a 0-5 scale indicating stronger limitation). Similar observation was made for the association with availability as a limiting factor in initiating IVIg (r = –0.35, *P* < .01, Fig 3B). In contrast, as shown in Figure 3C, no association was found with costs as a reason for discontinuation (rated from 1 = most important to 7 = least important) in terms of HDI (r = 0.06, *P* = .63). A positive correlation between HDI and availability as a reason for discontinuation was found (r = 0.26, *P* = .03, Fig 3D). Similar findings were observed when applying GDP per capita as a socioeconomic metric (Supplementary Fig S2).

## DISCUSSION

This multinational survey provides the first overview of real-world practices concerning IVIg therapy in IIM. Following the publication of the ProDERM trial, this study provides valuable insight into how IVIg is used across disease subtypes, organ manifestations, and healthcare systems.

Although high-dose corticosteroids and traditional immunosuppressants remain the foundation of IIM treatment [15], IVIg has gained increasing attention, particularly in complex clinical situations. Our findings confirm that IVIg is infrequently used as a first-line therapy but has a key role when conventional strategies fail or are contraindicated. Refractory disease, rapidly progressive course, and contraindications to other immunosuppressants were reported as the most frequent triggers for IVIg initiation. Severe muscular involvement, particularly with extramuscular complications such as dysphagia, was another major indication. In this context, IVIg was the most commonly reported first-line option for dysphagia, which emphasises its perceived efficacy and safety in neuromuscular swallowing dysfunction, even in the absence of dedicated clinical trials. Interestingly, our survey also sheds light on how IVIg is employed for manifestations beyond the ‘muscle’ aspect. Skin involvement, ILD, gastrointestinal, and cardiac complications were all noted as relevant indications, typically in the second or third line. These data reflect the often empirical use of IVIg in complex multi-organ presentations of IIM, where evidence remains sparse. In particular, the frequent use in ILD and CAM also shows a therapeutic gap in evidence-based guidance and the need for further studies to support these real-world practices. AEs were frequently reported, with headaches and infusion reactions among the most common. Serious complications such as thromboembolism and aseptic meningitis were also reported, which may reflect the need for careful risk assessment in real-world IVIg use. Interpretation of these findings requires caution, as AEs were reported at the centre level and do not reflect patient-level incidence rates, which limits direct comparison with randomised trials such as ProDERM [10]. However, the spectrum of reported events is in line with the known safety profile of IVIg.

Regionally, interesting differences were observed between European and non-European centres. Non-European sites more frequently used IVIg in ASyS, ILD, and antibody-positive patients and reported higher usage in the setting of infections and immunosuppressive contraindications. They were also more likely to have previously treated patients with cyclophosphamide or calcineurin/mTOR inhibitors-agents, which are typically reserved for more severe or refractory disease in European centres. By contrast, European sites more often employed methotrexate concomitantly with IVIg and reported greater reliance on national guidelines or reimbursement frameworks. These regional contrasts demonstrate how local drug availability, funding structures, and therapeutic traditions influence not just whether IVIg is used, but also in whom, how, and for how long. Perhaps the most policy-relevant aspect of our findings relates to the socioeconomic landscape in which IVIg treatment decisions are made. As an expensive therapy, IVIg is at the intersection of clinical need and financial feasibility. Our analysis, which linked centre-specific responses to HDI as well as national GDP per capita, revealed a significant association between socioeconomic strength and limitations in initiating IVIg. Specifically, cost and availability were reported as greater barriers in less affluent countries/regions. This suggests that in resource-limited health systems, IVIg may be rationed or deprioritised despite clinical indication, which may raise concerns about equitable access to care. However, once IVIg treatment is initiated, economic limitations appear to play a less important role in discontinuation decisions. Notably, there was no correlation between HDI or GDP and cost as a reason for stopping therapy, which may mean that once therapy is started, continuation is driven more by clinical response and patient need than by economics. The fact that IVIg use is influenced not only by disease severity or serological profile, but also by socioeconomic indicators like HDI and GDP, as well as structural factors such as institutional policies and regional drug access, echoes broader patterns of disparity described in rheumatological care. As highlighted in recent literature, health disparities in rheumatology are multifactorial and persistent, influencing not just drug access but also the availability of multidisciplinary services, health literacy, and treatment adherence. Therefore, our findings reflect growing awareness that treatment decisions are influenced by unequal healthcare systems. Addressing these gaps will require coordinated efforts and international guidance that considers socioeconomic and structural barriers to ensure fair access to care [14,[16], [17], [18]].

From a methodological perspective, the strength of this study lies in its wide international representation and structured approach. By addressing centres rather than individual physicians, we aimed to capture more institutional treatment paradigms. Moreover, the inclusion of rheumatologists and nonrheumatologists allowed for a holistic view of IVIg use across clinical disciplines, dedicated to treatment of patients with myositis. However, some limitations must be acknowledged. First, the survey relies on self-reported data, which carries a degree of subjectivity. Although participants were encouraged to report institutional rather than personal practices, the accuracy of certain items such as frequency of AEs, reasons for discontinuation, or estimation of serological profiles may vary depending on local documentation standards and recall bias. More than 70% of centres reported currently treating fewer than 10 patients with IVIg, indicating that IVIg experience at many centres is based on small patient numbers, which may further increase susceptibility to recall bias. In addition, because this study is based on physician-reported survey data rather than objective or registry-based prescribing data, the results reflect clinicians’ perceptions of institutional IVIg use. The findings should therefore be interpreted as reported practice patterns rather than precise measures. Another caveat concerns the classification of IIM subtypes, which was based on clinician-reported diagnoses rather than centrally adjudicated criteria. This introduces potential heterogeneity, particularly in regions where diagnostic work-up or access to antibody testing differ. In addition, the survey does not account for regional differences in the relative frequency of IIM subtypes or broader clinical heterogeneity across countries or regions, which may influence observed IVIg prescribing patterns independent of treatment preferences or availability. In some settings, outdated classification systems may still guide clinical diagnosis, treatment decisions, and IVIg eligibility. Moreover, our AE data, reported at the centre level, cannot be directly compared with patient-level safety outcomes from controlled trials such as ProDERM. Another important caveat is that some centres may have interpreted certain survey items differently. We attempted to clarify these through explicit instructions, but subtle variation in interpretation cannot be excluded.

In summary, this study provides granular and geographically diverse evidence of how IVIg is used in real-world myositis care. Our findings support the call for harmonised international guidance that not only reflects clinical indication but also promotes fairness and access across different socioeconomic settings. As IVIg continues to evolve from an empiric option to an evidence-supported remedy in IIM care, this global survey provides a timely insight into ongoing practice patterns worldwide.

## Appendix Supplementary materials

Image, application 1

Image, application 2

## Footnotes

Supplementary material associated with this article can be found in the online version at doi:10.1016/j.ero.2026.02.012.
