# Supplementary material for: International IVIg prescription patterns in idiopathic inflammatory myopathies: real-world insights from the MyoNet survey
Source: EULAR Rheumatol Open. 2026 Mar 3;2(1):289–96. doi: 10.1016/j.ero.2026.02.012 (PMC13292478; doi:10.1016/j.ero.2026.02.012)
Supplement: Supplementary file 1 [file mmc1.pdf]

International IVIg prescription patterns in idiopathic inflammatory myopathies: real-world insights from the MyoNet survey

Online Supplementary appendix

Table of contents

Table S1: Characteristics of participating centers ……………………………………………………………………………3

Figure S1: Distribution of idiopathic inflammatory myopathy (IIM) subtypes treated with intravenous immunoglobulin (IVIg) across participating centers……………………………………………………………………..5

Table S2: Estimated clinical characteristics of IIM patients receiving IVIg therapy, as reported by participating centers…….…6

Table S3: Overview of prior and concomitant immunosuppressive treatments in patients receiving IVIg.…….…7

Table S4. Clinical scenarios and disease severity grades reported as relevant considerations for IVIg use in IIM……….…8

Table S5. Practices related to IVIg dosing, regimen interval, treatment duration, subcutaneous usage, treatment modifications, and response evaluation across participating centres (n = 68)…………9

Table S6. Regional differences in IVIg practices among European and non-European centres.………….10

Figure S2: Correlation between national GDP and perceived barriers to IVIg use…………………………….15

Characteristics of the centres	n	%
**Number of centres**	68	100.0
**Number of IIM patients**		
< 10	5	7.4
10-25	9	13.2
26-50	18	26.5
51-100	16	23.5
101-150	8	11.8
> 150	0	0.0
**Number of patients currently receiving IVIg**		
< 10	49	72.1
10-25	16	23.5
26-50	2	2.9
> 50	0	0.0
**Country/Region**		
Austria	7	10.3
Italy	6	8.8
United Kingdom	6	8.8
China	5	7.4
Hong Kong	5	7.4
Sweden	4	5.9
Belgium	3	4.4
France	3	4.4
Netherlands	3	4.4
Singapore	3	4.4
Spain	3	4.4
Argentina	2	2.9
Denmark	2	2.9
Germany	2	2.9
Portugal	2	2.9
Brazil	1	1.5
Chile	1	1.5
Finland	1	1.5
Hungary	1	1.5
Israel	1	1.5
Lithuania	1	1.5
Norway	1	1.5
Poland	1	1.5
Romania	1	1.5
South Korea	1	1.5
Taiwan	1	1.5
Vietnam	1	1.5
**Specialty**		
Rheumatology	49	72.1
Internal medicine	10	14.7
Neurology	4	5.9
Pediatrics	3	4.4
Dermatology	1	1.5
Pulmonology	1	1.5
**Years practicing**		

< 5 years	4	5.9
5-10 years	14	20.6
11-20 years	28	41.2
> 20 years	18	26.5

Table S1. Characteristics of participating centers. Overview of characteristics including medical specialty, years of professional experience, geographic distribution, and number of IIM patients under care as well as patients currently receiving IVIg therapy. The table presents data from 68 centers in 27 countries with complete or near-complete survey submissions (after excluding responses with high level of missingness). **Abbreviations**: n, number of centres; % (percentage) proportion of centres; IIM: idiopathic inflammatory myopathy; IVIg: intravenous immunoglobulin.

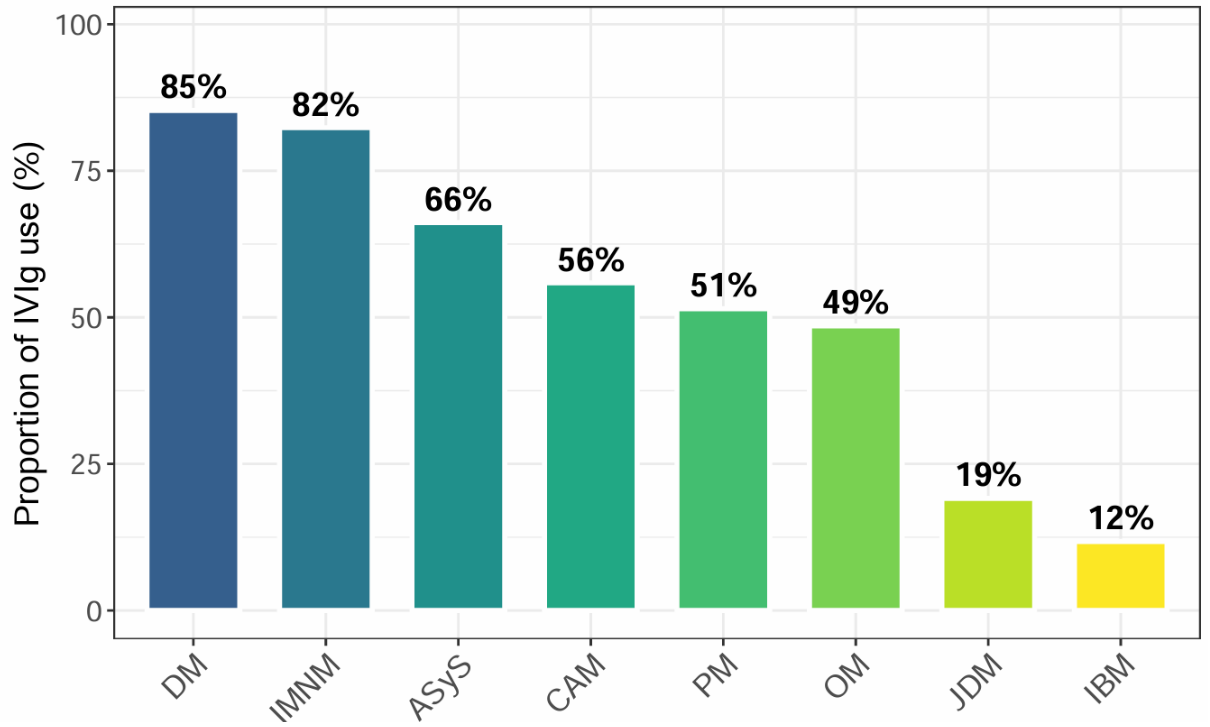

Figure S1. Distribution of idiopathic inflammatory myopathy (IIM) subtypes treated with intravenous immunoglobulin (IVIg) across participating centers. Absolute numbers and corresponding percentages of centers reporting IVIg use for each subtype are as follows: dermatomyositis (DM) 58 centers (85.3%); immune-mediated necrotizing myopathy (IMNM) 56 centers (82.4%); antisynthetase syndrome (ASyS) 45 centers (66.2%); cancer-associated myositis (CAM) 38 centers (55.9%); polymyositis (PM) 35 centers (51.5%); overlap myositis (OM) 33 centers (48.5%); juvenile dermatomyositis (JDM) 13 centers (19.1%); and inclusion body myositis (IBM) 8 centers (11.8%). **Abbreviations:** DM, dermatomyositis; IMNM, immune-mediated necrotizing myopathy; ASyS, antisynthetase syndrome; CAM, cancer-associated myositis; PM, polymyositis; OM, overlap myositis; JDM, juvenile dermatomyositis; IBM, inclusion body myositis.

Clinical profile of patients receiving IVIg therapy	Mean ± SD
Positive for MSA and/or MAA	73.4 ± 29.8
With severe muscle involvement	58.8 ± 33.5
Refractory to other immunosuppressants	49.8 ± 32.7
With rapidly progressive disease	35.4 ± 29.7
With contraindications to other therapies	27.1 ± 28.8
With severe skin involvement	24.9 ± 25.1
With Interstitial Lung Disease (ILD)	23.6 ± 23.4
Negative for Myositis-Specific Antibodies (MSA) and/or Myositis-Associated Antibodies (MAA)	18.5 ± 21.4

Table S2. Estimated clinical characteristics of IIM patients receiving IVIg therapy, as reported by participating centers. Respondents/Centres were asked to estimate the proportion of their IVIg-treated IIM patients who met each clinical criterion. Reported values represent the mean ± standard deviation (SD) of these center-level estimates. **Abbreviations:** MSA, myositis-specific antibodies; MAA, myositis-associated antibodies; ILD, interstitial lung disease; SD, standard deviation.

DMARD failures before IVIg initiation	n	%
1	16	23.5
2	20	29.4
3	9	13.2
4	2	2.9
5	0	0.0
>5	0	0.0
**DMARDs commonly used prior to IVIg**		
Methotrexate	55	80.9
Mycophenolate -Mofetil	47	69.1
Rituximab	32	47.1
Azathioprine	30	44.1
Ciclosporin, Tacrolimus, Everolimus, Sirolimus	20	29.4
Cyclophosphamide	20	29.4
Leflunomide	4	5.9
Tocilizumab	2	2.9
Sulfasalazine	0	0.0
Other (n=2 JAKi, n=1 HCQ, n=1 Anifrolumab)	4	5.9
**DMARDs used concurrently with IVIg**		
Glucocorticoids	60	88.2
Methotrexate	54	79.4
Mycophenolate mofetil	48	70.6
Rituximab	33	48.5
Azathioprine	27	39.7
Ciclosporin, Tacrolimus, Everolimus, Sirolimus	27	39.7
Cyclophosphamide	16	23.5
Tocilizumab	5	7.4
Leflunomide	3	4.4
Sulfasalazine	1	1.5
Other (n=2 Tofacitinib, n=1 Anakinra, n=1 HCQ)	4	5.9
**Proportion of patients with IVIg as first line treatment (Mean ± SD)**	22.1 ± 22.4	
**Proportion of patients with IVIg as first line treatment (Median; IQR)**	15.0 (5.0; 30.0)	
**Proportion of patients with concomitant immunosuppressive treatment in addition to IVIg (Mean ± SD)**	80.7 ± 24.5	

Table S3: Overview of prior and concomitant immunosuppressive treatments in patients receiving IVIg. The table summarizes how many disease-modifying antirheumatic drugs (DMARDs) had been applied before IVIg initiation and lists the most frequently employed agents both prior to and during IVIg therapy, as reported by participating centers. Additionally, the mean proportion (± standard deviation) of patients for whom IVIg was initiated as first-line treatment and the average proportion of patients receiving any concomitant immunosuppressive therapy are shown (based on center-level estimates obtained via percentage bar slider). **Abbreviations:** IVIg, intravenous immunoglobulin; n, number of centres; % (percentage) proportion of centres/responses; DMARDs, disease-modifying antirheumatic drugs; JAKi, Janus kinase inhibitor; HCQ, hydroxychloroquine; SD, standard deviation; IQR, interquartile range.

**IVIg treatment considerations based on clinical circumstances**	**n**	**%**
Refractory to other immunosuppressants	60	88.2
Rapidly progressive disease	52	76.5
Dysphagia	50	73.5
Contraindications to other therapies	46	67.6
Immune-Mediated Necrotizing Myopathy (IMNM)	43	63.2
Concomitant infection	37	54.4
Concomitant / Cancer Associated Myositis	36	52.9
Conception, Pregnancy, Breast feeding	19	27.9
Steroid sparing agent	18	26.5
**Disease severity grades considered when initiating IVIg therapy**		
Mild	3	4.4
Moderate	30	44.1
Severe	35	51.5

Table S4. Clinical scenarios and disease severity grades reported as relevant considerations for IVIg use in IIM. Values represent the number and percentage of centers (n= 68) selecting each circumstance or severity category as applicable. **Abbreviations:** IVIg, intravenous immunoglobulin; n, number of centres; % (percentage) proportion of centres/responses; IMNM, immune-mediated necrotizing myopathy.

**Body weight reference used for IVIg dosing calculation**	**n**	**%**
Actual body weight	43	63.2
Ideal body weight	19	27.9
Unsure	3	4.4
**IVIg dosage used**		
0.5 g/kg body weight	2	2.9
1 g/kg body weight	10	14.7
2 g/kg body weight	51	75.0
Other (0.4 g/kg body weight)	2	2.9
**Average interval between IVIg cycles**		
4 weeks	56	82.4
5 weeks	1	1.5
6 weeks	2	2.9
Other (n=1 2 weeks, n=1 3 weeks, n=1 8 weeks)	3	4.4
**Average duration for a course of IVIg (months, mean ± SD)**	5.9 ± 5.2	
**Average duration for a course of IVIg (months, median, IQR)**	4.5 (3.0; 6.0)	
**Time efficacy assessment**		
6 weeks	28	41.2
12 weeks	35	51.5
24 weeks	3	4.4
36 weeks	1	1.5
Other (n=4 4 weeks, n=1 <4 weeks, n=1 16 weeks)	6	8.8
**Modifications after disease control**		
Extend regimen interval	29	42.6
Extend regimen interval and reduce dose of IVIg	17	25.0
Reduce dose of IVIg by 50%	14	20.6
Maintain starting dose and initial regimen interval	12	17.6
Stop	11	16.2
Reduce dose of IVIg by 25%	5	7.4
**Subcutaneous immunoglobulin (SCIg)**	10	14.7
**Measures to evaluate response to IVIg; 0-5 rating scale (0 = not assessed, 5 = integral component of monitoring; mean ± SD)**		
ACR / EULAR Total Improvement Score	1.9 ± 1.8	
Patient-reported outcomes	3.4 ± 1.5	
Muscle enzymes	4.5 ± 0.8	
Reduction in steroid dose	4.1 ± 1.2	
Timed assessments	1.9 ± 1.6	

Table S5. Practices related to IVIg dosing, regimen interval, treatment duration, subcutaneous usage, treatment modifications, and response evaluation across participating centres (n = 68). Values indicate the number and percentage of centres using each strategy. Measurement opportunities to evaluate response were rated on a 0-5 rating scale (0= not assessed/relevant, 5= integral component of response monitoring). **Abbreviations:** IVIg, intravenous immunoglobulin; ACR, American College of Rheumatology; EULAR, European Alliance of Associations for Rheumatology; SD, standard deviation; IQR, interquartile range.

	European	Other Regions	p-value
**Number of centres n (%)**	47 (100%)	21 (100%)	
**IIM Subtypes treated with IVIg**			
Dermatomyositis (DM)	83.0%	90.5%	0.73
Immune-Mediated Necrotizing Myopathy (IMNM)	80.9%	85.7%	0.89
Antisynthetase Syndrome (ASyS)	57.4%	85.7%	0.04
Cancer associated Myositits (CAM)	53.2%	61.9%	0.69
Polymyositis (PM)	48.9%	57.1%	0.72
Overlap Myositis (OM)	53.2%	38.1%	0.37
Juvenile Dermatomyositis (JDM)	21.3%	14.3%	0.73
Inclusion Body Myositis (IBM)	12.8%	9.5%	1.00
**Clinical Profile of Patients on IVIg Therapy (%; mean ± SD)**			
Positive for MSA and/or MAA	70.0 ± 30.3	80.8 ± 27.9	0.07
With severe muscle involvement	59.2 ± 34.9	58.2 ± 31.2	0.79
Refractory to other immunosuppressants	46.0 ± 31.0	58.5 ± 35.7	0.20
With rapidly progressive disease	32.5 ± 28.2	41.5 ± 32.8	0.34
With contraindications to other therapies	19.4 ± 23.7	42.2 ± 32.5	0.01
With severe skin involvement	23.7 ± 23.6	27.9 ± 29.0	0.72
With Interstitial Lung Disease (ILD)	20.1 ± 20.5	31.5 ± 28.2	0.13
Negative for Myositis-Specific Antibodies (MSA) and/or Myositis-Associated Antibodies (MAA)	19.9 ± 19.7	15.6 ± 25.1	0.14
**IVIg in first line (%)**	24.0 ± 25.0	17.6 ± 14.0	0.78
**Disease severity grades considered when initiating IVIg therapy**			
Mild	6.4%	0.0%	0.55
Moderate	44.7%	54.4%	0.75
Severe	91.4%	95.2%	1.00
**IVIg treatment considerations based on clinical circumstances**			
Refractory to other immunosuppressants	91.5%	81.0%	0.24
Rapidly progressive disease	74.5%	81.0%	0.76
Dysphagia	74.5%	71.4%	1.00
Contraindications to other therapies	68.1%	66.7%	1.00
Immune-Mediated Necrotizing Myopathy (IMNM)	55.3%	81.0%	0.08
Concomitant infection	44.7%	76.2%	0.03
Concomitant / Cancer Associated Myositis	46.8%	66.7%	0.21
Conception, Pregnancy, Breast feeding	29.8%	23.8%	0.83
Steroid sparing agents	27.7%	23.8%	0.97

**IVIg use by clinical manifestation and treatment line (% of centres)**			
Muscle involvement			0.55
First line	22.2	19.0	
Second line	46.7	52.4	
Third line	31.1	23.8	
Not considered	0.0	4.8	
Lung involvement			1.00
First line	9.1	9.5	
Second line	31.8	33.3	
Third line	43.2	42.9	
Not considered	15.9	14.3	
Skin involvement			0.31
First line	8.9	0.0	
Second line	55.6	55.0	
Third line	28.9	25.0	
Not considered	6.7	20.0	
Joint involvement			0.91
First line	72.7	70.0	
Second line	6.8	10.0	
Third line	20.5	20.0	
Not considered	0.0	0.0	
Heart involvement			0.78
First line	18.6	9.5	
Second line	32.6	38.1	
Third line	25.6	33.3	
Not considered	23.3	19.0	
Gastrointestinal involvement			0.36
First line	13.6	4.8	
Second line	20.5	33.3	
Third line	27.3	38.1	
Not considered	38.6	23.8	
Dysphagia			0.56
First line	51.1	45.0	
Second line	33.3	50.0	
Third line	13.3	5.0	
Not considered	2.2	0.0	
**DMARD failures before IVIg initiation**			0.57
1	29.5%	23.8%	
2	25.0%	23.8%	
3	29.5%	33.3%	
4	13.6%	14.3%	
5	2.3%	4.8%	
>5	0.0%	0.0%	
**DMARDs commonly used prior to IVIg**			
Methotrexate	85.1%	71.4%	0.20
Mycophenolate -Mofetil	66.0%	76.2%	0.57
Rituximab	46.8%	47.6%	1.00
Azathioprine	36.2%	61.9%	0.07

Ciclosporin, Tacrolimus, Everolimus, Sirolimus	12.8%	66.7%	<0.01
Cyclophosphamide	21.3%	47.6%	0.04
Leflunomide	4.3%	9.5%	0.58
Tocilizumab	2.1%	4.8%	0.53
Sulfasalazine	0.0%	0.0%	1.00
Other (n=2 JAKi, n=1 HCQ, n=1 Anifrolumab)	4.3%	4.8%	0.58
**Body weight reference used for IVIg dosing calculation**			0.10
Actual body weight	61.4%	76.2%	
Ideal body weight	36.4%	14.2%	
Unsure	2.3%	9.5%	
**IVIg dosage used**			0.58
0.5 g/kg body weight	2.3%	4.8%	
1 g/kg body weight	18.2%	9.5%	
2 g/kg body weight	77.3%	81.0%	
Other (0.4 g/kg body weight)	2.3%	4.8%	
**Average interval between IVIg cycles**			0.61
4 weeks	80.4%	100.0%	
5 weeks	2.2%	0.0%	
6 weeks	4.3%	0.0%	
Other (n=1 2 weeks, n=1 3 weeks, n=1 8 weeks)	6.5%	0.0%	
**Limitations in administering IVIg (rating scale from 0= not a limitation to 5 =major limitation; mean ± SD)**			
Costs	2.7 ± 1.9	4.0 ± 1.4	<0.01
Availability	2.2 ± 2.0	1.0 ±1.5	0.02
Authorities	2.3 ± 1.9	2.0 ± 1.8	0.51
Patients' preferences	0.9 ± 1.1	1.7 ± 1.3	0.01
Side effects	1.0 ± 0.9	1.4 ± 1.1	0.10
Lack of infusion facilities	0.8 ± 1.2	1.0 ± 1.7	0.95
**Measures to evaluate response to IVIg; 0-5 rating scale (0= not assessed, 5= integral component of monitoring; mean ± SD)**			
ACR / EULAR Total Improvement Score	2.2 ± 1.9	1.4 ± 1.6	0.11
Patient-reported outcomes	3.6 ± 1.4	3.1 ± 1.7	0.25
Muscle enzymes	4.4 ± 0.8	4.8 ± 0.6	0.04
Reduction in steroid dose	4.0 ± 1.2	4.3 ± 1.1	0.43
Timed assessments	1.9 ± 1.5	2.1 ± 1.8	0.80
**Patients' concomitant treatment in addition to IVIg (%)**	81.5 ± 22.3	79.0 ± 29.0	0.89
**Agents used concurrently with IVIg**			
Glucocorticoids	87.2%	90.5%	1.00
Methotrexate	87.2%	61.9%	0.02
Mycophenolate mofetil	70.2%	71.4%	1.00
Rituximab	51.0%	42.9%	0.72
Azathioprine	38.3%	42.9%	0.93
Ciclosporin, Tacrolimus, Everolimus, Sirolimus	27.7%	66.7%	<0.01
Cyclophosphamide	21.3%	28.6%	0.55
Tocilizumab	6.4%	9.5%	0.64

Leflunomide	4.3%	4.8%	1.00
Sulfasalazine	2.1%	0.0%	1.00
Other (n=2 Tofacitinib, n=1 Anakinra, n=1 Hydroxychloroquine)	6.4%	4.8%	1.00
**Adverse events**	83.0%	61.9%	0.07
**Adverse events specified**			
Headaches	72.3%	38.1%	0.02
Infusion reaction (allergic, anaphylactic)	34.0%	61.9%	0.06
Fever	31.9%	38.1%	0.83
Nausea	27.7%	23.8%	0.97
Thromboembolism (Deep -vein thrombosis, Pulmonary embolism)	23.4%	23.8%	1.00
Aseptic Meningitis	25.5%	14.3%	0.36
Chills	19.1%	19.0%	1.00
Myalgia	12.8%	19.0%	0.49
Vomiting	8.5%	9.5%	1.00
Cerebrovascular accident/Cerebral infarction	4.3%	4.8%	1.00
Other (n=2 myocardial infarction, n=1 serum sickness, n=1 other immune disease, n=1 neutropenia)	8.5%	4.8%	1.00
**Average duration for a course of IVIg (months)**	6.5 ± 5.8	4.6 ± 3.6	0.20
**Time efficacy assessment**			
6 weeks	34.0%	57.1%	0.13
12 weeks	57.4%	38.1%	0.23
24 weeks	6.4%	4.8%	1.00
36 weeks	2.1%	0.0%	1.00
Other (n=4 4 weeks, n=1 <4 weeks, n=1 16 weeks)	6.4%	14.3%	0.36
**Modifications Post Disease Control**			
Extend regimen interval	42.6%	42.9%	0.88
Extend regimen interval and reduce dose of IVIg	23.4%	28.6%	1.00
Reduce dose of IVIg by 50%	29.8%	4.8%	0.03
Maintain starting dose and initial regimen interval	6.4%	42.9%	<0.01
Stop	17.0%	14.3%	1.00
Reduce dose of IVIg by 25%	6.4%	9.5%	0.64
**Reason for discontinuation (rating scale from 1= most important to 7= least important; mean ± SD)**			
Disease remission	1.7 ± 1.0	2.5 ± 1.9	0.16
Lack of efficacy	2.4 ± 1.6	3.0 ± 1.9	0.10
Costs	3.8 ± 1.3	2.6 ± 1.4	<0.01
Patients' preference	4.4 ± 1.4	4.1 ± 1.2	0.52
Authorities	4.5 ± 1.6	4.3 ± 1.6	0.58
Availability	4.9 ± 1.5	5.3 ± 1.5	0.14
Lack of infusion facillities	6.3 ± 1.3	6.0 ± 1.7	0.41
**Subcutaneous immunoglobulin (SCIg) (yes)**	21.3%	0.0%	0.03
**National guidelines or commissioning criteria policies**	40.9%	4.8%	<0.01

Table S6. Regional differences in IVIg practices among European and non-European centres. Data are presented as percentages or mean ± standard deviation unless otherwise indicated. P-values reflect comparisons between groups (Europe vs. other Regions). Frequencies are based on total responses per group (Europe: n = 47; Other Regions: n = 21). **Abbreviations:** IVIg: Intravenous immunoglobulin; IIM: Idiopathic inflammatory myopathies; DM: Dermatomyositis; IMNM: Immune-mediated necrotizing myopathy; ASyS: Antisynthetase syndrome; CAM: Cancer-associated myositis; PM: Polymyositis; OM: Overlap myositis; JDM: Juvenile dermatomyositis; IBM: Inclusion body myositis; MSA: Myositis-specific antibodies; MAA: Myositis-associated antibodies; ILD: Interstitial lung disease; DMARD: Disease-modifying antirheumatic drug; JAKi: Janus kinase inhibitor; HCQ: Hydroxychloroquine; SCIg: Subcutaneous immunoglobulin; ACR: American College of Rheumatology; EULAR: European Alliance of Associations for Rheumatology; SD: Standard deviation. **Scales used:** Limitations in IVIg administration: 0= no limitation to 5= major limitation; Measures to evaluate response to IVIg: 0= not assessed to 5= integral component of monitoring; Reason for discontinuation: 1= most important to 7= least important.

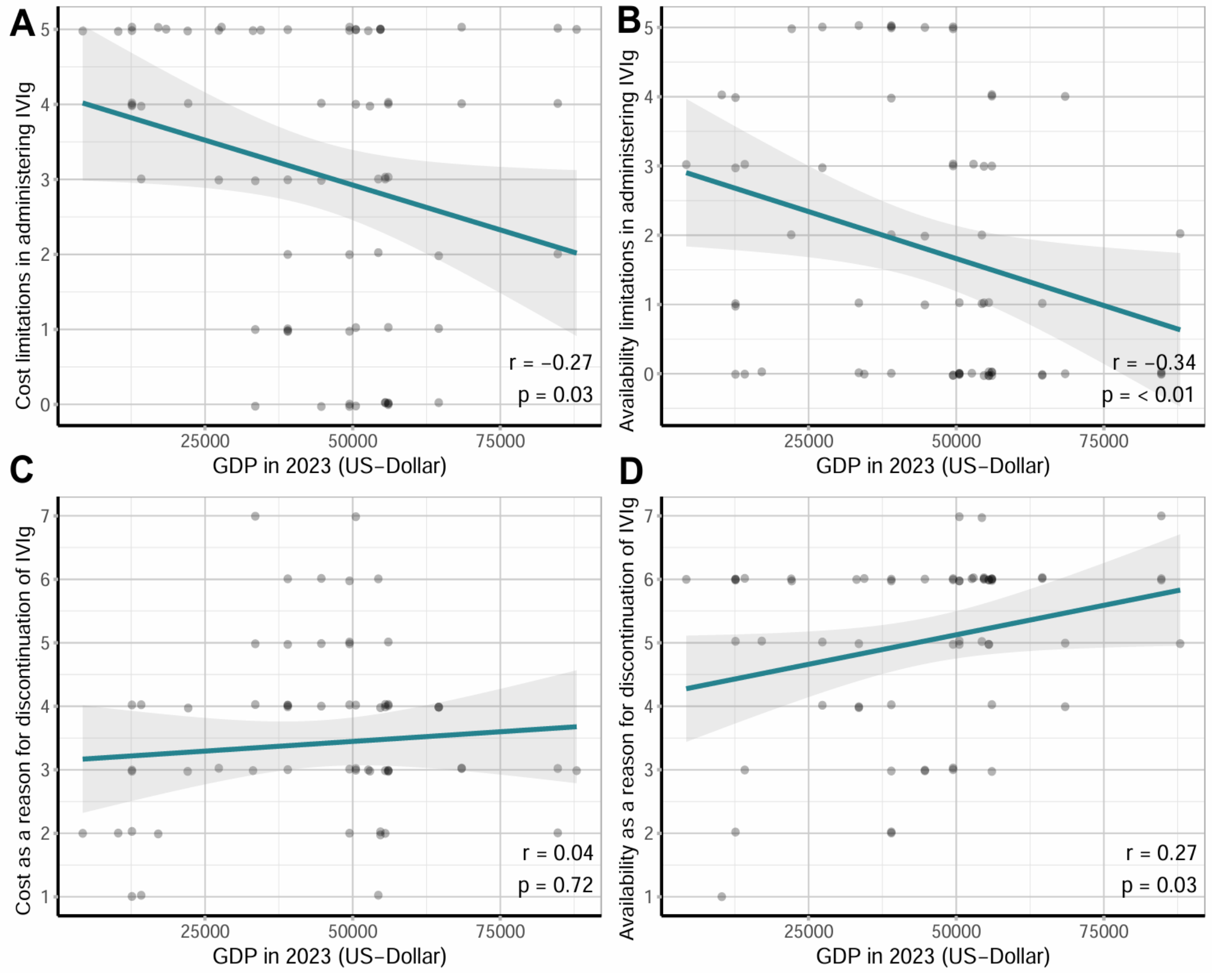

Figure S2: Correlation between national GDP and perceived barriers to IVIg use. Spearman's rank correlation coefficients (r) and p-values (p) are shown; lower scores indicate greater limitation or higher importance. Panel A: Cost as a limitation to initiating IVIg (rating 0-5; r=-0.27, p=0.03); Panel B: Availability as a limitation to initiating IVIg (rating 0-5; r=-0.34, p<0.01); Panel C: Cost as a reason for discontinuation (rating 1-7; r=0.04, p=0.72); Panel D: Availability as a reason for discontinuation (rating 1-7; r=0.27, p=0.03). Rating scales: 0-5= limitation severity (0= none, 5= major) for Panels A/B; 1-7= importance of discontinuation reason (1= most, 7= least) for Panels C/D. **Abbreviations:** IVIg: Intravenous immunoglobulin; GDP: Gross Domestic Product; Spearman's r: Spearman rank correlation coefficient; p: p-value.
