# Supplementary material for: International IVIg prescription patterns in idiopathic inflammatory myopathies: real-world insights from the MyoNet survey
Source: EULAR Rheumatol Open. 2026 Mar 3;2(1):289–96. doi: 10.1016/j.ero.2026.02.012 (PMC13292478; doi:10.1016/j.ero.2026.02.012)
Supplement: Supplementary file 2 [file mmc2.pdf]

MYONET

Intravenous immunoglobulin (IVIg) treatment in patients with idiopathic inflammatory myopathies (IIM)- a survey

1. How many IIM patients are under your care (not limited to IVIg therapy)?

◯ < 10

◯ 10-25

◯ 26-50

◯ 51-100

◯ 101-150

◯ >150

2. How many IIM patients are **currently receiving IVIg** treatment within your cohort?

◯ < 10

◯ 10-25

◯ 26-50

◯ >50

MYONET

Intravenous immunoglobulin (IVIg) treatment in patients with idiopathic inflammatory myopathies (IIM)- a survey

Subtypes of IIM treated

3. Which subtypes of IIM do you treat with IVIg at your centre? (Please select all that apply)

- ☐ Dermatomyositis (DM)
- ☐ Juvenile Dermatomyositis (JDM)
- ☐ Antisynthetase Syndrome (ASyS)
- ☐ Immune-Mediated Necrotizing Myopathy (IMNM)
- ☐ Inclusion Body Myositis (IBM)
- ☐ Overlap Myositis (OM)
- ☐ Cancer associated Myositits (CAM)
- ☐ Polymyositis (PM)
- ☐ Other (please specify)

4. Of the patients **currently receiving IVIg** therapy, what is the approximate percentage fulfilling following categories?

% Negative for Myositis-Specific Antibodies (MSA) and/or Myositis-Associated Antibodies (MAA)

% Positive for MSA and/or MAA

% With Interstitial Lung Disease (ILD)

% With severe muscle involvement

% With sever skin involvement

% Refractory to other immunosuppressants

% With rapidly progressive disease

% With contraindications to other therapies

5. Of your patients with IIM, what percentage have received IVIg as **first line** treatment?

0 50 100

6. When considering **IVIg therapy** for your IIM patients, which **disease severity grades** do you consider for your treatment decision? (Please select all that apply)

- [ ] Mild
- [ ] Moderate
- [ ] Severe
- [ ] Other (please specify)

7. When considering **IVIg therapy** for your IIM patients, which **clinical circumstances** influence your treatment decision? (Please select all that apply)

- ☐ Refractory to other immunosuppressants
- ☐ Rapidly progressive disease
- ☐ Contraindications to other therapies
- ☐ Steroid sparing agents
- ☐ Dysphagia
- ☐ Immune-Mediated Necrotizing Myopathy (IMNM)
- ☐ Concomitant / Cancer Associated Myositis
- ☐ Conception, Pregnancy, Breast feeding
- ☐ Concomitant Infection
- ☐ Other (please specify)

8. Please indicate the importance of **IVIg therapy** for each of the listed clinical IIM **manifestations or scenarios** according to your clinical practice.

	First line	Second line	Third line	Not considered
Muscle involvement	○	○	○	○
Lung involvement	○	○	○	○
Skin involvement	○	○	○	○
Joint involvement	○	○	○	○
Heart involvement	○	○	○	○
Gastrointestinal involvement	○	○	○	○
Dysphagia	○	○	○	○

MYONET

Intravenous immunoglobulin (IVIg) treatment in patients with

9. If IVIg treatment is used in refractory patients, how many DMARDs and / or biologics should the patient have failed **before initiating IVIg**?

- ◯ 1
- ◯ 2
- ◯ 3
- ◯ 4
- ◯ 5
- ◯ > 5
- ◯ Do DMARDs used in your practice include biologics? (Yes/No)

10. In refractory patients, which DMARDs have you usually used **prior to initiating IVIg** treatment?

- ☐ Methotrexate
- ☐ Azathioprine
- ☐ Leflunomide
- ☐ Sulfasalazine
- ☐ Mycophenolate -Mofetil
- ☐ Ciclosporin, Tacrolimus, Everolimus, Sirolimus
- ☐ Rituximab
- ☐ Tocilizumab
- ☐ Cyclophosphamide
- ☐ Other (please specify)

11. Do you use ideal or actual body weight to calculate IVIg dosage?

- ◯ Ideal body weight
- ◯ Actual body weight
- ◯ Unsure
- ◯ Other (please specify)

12. What is the usual starting dosage of an IVIg cycle in your practice?

- ◯ 0.5 g/kg body weight
- ◯ 1 g/kg body weight
- ◯ 2 g/kg body weight
- ◯ Other (please specify)

13. What is the average interval between IVIg cycles in your practice? (in weeks)

- ◯ 3 weeks
- ◯ 4 weeks
- ◯ 5 weeks
- ◯ 6 weeks
- ◯ Other (please specify)

14. What limitations or challenges do you face in administering IVIg to patients with IIM? (0 no limitation to 5 major limitation)

	0	1	2	3	4	5
Costs	◯	◯	◯	◯	◯	◯
Availability	◯	◯	◯	◯	◯	◯
Authorities (i.e. governmental, institutional or stewardship)	◯	◯	◯	◯	◯	◯
Patients' preferences	◯	◯	◯	◯	◯	◯
Side effects	◯	◯	◯	◯	◯	◯
Lack of infusion facilities	◯	◯	◯	◯	◯	◯

Other (please specify)

15. Which of the following do you use to evaluate the response of patients to IVIG therapy? (0 not used/assessed to 5 integral component)

	0	1	2	3	4	5
ACR / EULAR Total Improvement Score	◯	◯	◯	◯	◯	◯
Patient-reported outcomes	◯	◯	◯	◯	◯	◯
Muscle enzymes (CK, ALT [SGLT], AST [SGOT], Aldolase, Myoglobin)	◯	◯	◯	◯	◯	◯
Reduction in steroid dose	◯	◯	◯	◯	◯	◯
Timed assessment, such as six-minute walk test	◯	◯	◯	◯	◯	◯

Other (please specify)

16. What is the proportion of patients receiving concomitant immunosuppressive treatment in addition to IVIg?

0 50 100

17. Which of the following agents do you use as a concomitant therapy to IVIg?

- ☐ Glucocorticoids
- ☐ Methotrexate
- ☐ Azathioprine
- ☐ Leflunomide
- ☐ Sulfasalazine
- ☐ Mycophenolate -Mofetil
- ☐ Ciclosporin, Tacrolimus, Everolimus, Sirolimus
- ☐ Rituximab
- ☐ Tocilizumab
- ☐ Cyclophosphamide
- ☐ Other (please specify)

18. Have you observed any adverse events associated with IVIg therapy in IIM patients?

- ◯ Yes
- ◯ No

19. Which adverse events have you observed with IVIg? (Please select all that apply)

- [ ] Headaches
- [ ] Fever
- [ ] Nausea
- [ ] Vomiting
- [ ] Chills
- [ ] Myalgia
- [ ] Infusion reaction (allergic, anaphylactic)
- [ ] Thromboembolism (Deep -vein thrombosis, Pulmonary embolism)
- [ ] Cerebrovascular accident/Cerebral infarction
- [ ] Aseptic Meningitis
- [ ] Other (please specify)

20. What is the average duration in months for a course of IVIg in your practice?

21. How many weeks after IVIg initiation do you usually assess efficacy of treatment?

- [ ] 6 weeks
- [ ] 12 weeks
- [ ] 24 weeks
- [ ] 36 weeks
- [ ] Other (please specify)

22. Once disease control is achieved, do you change the dosing regimen (ie tapering dose or increase intervals)? Please tick all that apply. Please use free text at end to document your institutes protocol for dose tapering (if any).

- ☐ Maintain starting dose and initial regimen interval
- ☐ Extend regimen interval
- ☐ Reduce dose of IVIg by 25%
- ☐ Reduce dose of IVIg by 50%
- ☐ Extend regimen interval and reduce dose of IVIg
- ☐ Other (please specify)

23. What is the reason for discontinuation of IVIG therapy in your IIM patients? Please rank according to importance from 1 (most important) to 7 (least important).

- Disease remission
- Lack of efficacy
- Costs
- Patients' preference
- Authorities (i.e. governmental, institutional or stewardship)
- Availability
- Lack of infusion facilities

24. Do you utilize subcutaneous immunoglobulin (SCIg) therapy for IIM?

- ◯ Yes
- ◯ No

25. Are there any national guidelines or commissioning criteria policies in your country regarding the use of IVIg in IIM ?

- [ ] Yes
- [ ] No

26. You may upload the document directly through this survey platform, copy and paste the policy into the field provided below or contact us via email.

Choose File Choose File No file chosen

27. Please use this space to copy and paste the policy directly or include a weblink to specific guidelines.

MYONET

Intravenous immunoglobulin (IVIg) treatment in patients with idiopathic inflammatory myopathies (IIM)- a survey

Participant demographics

28. In which country do you practice?

29. What is your medical specialty?

- ☐ Rheumatology
- ☐ Neurology
- ☐ Dermatology
- ☐ Internal medicine
- ☐ Pulmonology
- ☐ Other (please specify)

30. How many years have you been practicing within your area of specialization, including years in training?

- ◯ < 5 years
- ◯ 5-10 years
- ◯ 11-20 years
- ◯ > 20 years

MYONET

Intravenous immunoglobulin (IVIg) treatment in patients with idiopathic inflammatory myopathies (IIM)- a survey

You have answered all questions - many thanks for your participation!

31. We welcome your feedback on the survey or any additional information you'd like to share.
